# Anti-Oxidized LDL Antibodies and Coronary Artery Disease: A Systematic Review

**DOI:** 10.3390/antiox8100484

**Published:** 2019-10-15

**Authors:** Victor J. van den Berg, Maxime M. Vroegindewey, Isabella Kardys, Eric Boersma, Dorian Haskard, Adam Hartley, Ramzi Khamis

**Affiliations:** 1 Department of Cardiology, Erasmus MC, University Medical Center Rotterdam, 3015 GD Rotterdam, The Netherlands; v.vandenberg@erasmusmc.nl (V.J.v.d.B.); m.vroegindewey@erasmusmc.nl (M.M.V.); i.kardys@erasmusmc.nl (I.K.); h.boersma@erasmusmc.nl (E.B.); 2 National Heart and Lung Institute, Imperial College London, W12 0NN London, UK; d.haskard@imperial.ac.uk (D.H.); adam.hartley12@imperial.ac.uk (A.H.)

**Keywords:** myocardial infarction, inflammation, antibody, coronary angiography, coronary artery disease

## Abstract

Antibodies to oxidized LDL (oxLDL) may be associated with improved outcomes in cardiovascular disease. However, analysis is restricted by heterogenous study design and endpoints. Our objective was to conduct a comprehensive systematic review assessing anti-oxLDL antibodies in relation to coronary artery disease (CAD). Through a systematic literature search, we identified all studies assessing the relationship of either, IgG or IgM ox-LDL/ copper-oxLDL/ malondialdehyde-LDL, with coronary atherosclerosis or cardiovascular events in populations with, and without, established CAD. Systematic review best practices were adhered to and study quality was assessed. An initial electronic database search identified 2059 records, which was subsequently followed by abstract and full-text review. Finally, we included 18 studies with over 1811 patients with CAD. The studies varied according to populations studied, conventional cardiovascular risk factors and interventional modalities used to assess CAD. IgM anti-oxLDL antibodies were found to indicate protection from more severe CAD and possibly cardiovascular events, whilst the relationship with IgG is more complex and difficult to elucidate, with studies reporting divergent results. In this systematic review, there is evidence that suggests a relationship between anti-oxLDL antibodies and CAD, especially for the IgM subclass. However, further studies, with well-characterized prospective cohorts, will be important to clarify these associations.

## 1. Introduction

Cardiovascular disease (CVD) remains the leading cause of death worldwide for the last 15 years [1], despite significant pharmaceutical and technological advancements. There is, therefore, a clear mandate for better, and earlier, identification of patients at risk, as well as improved management of CVD when it occurs. Oxidized low density lipoprotein (oxLDL) is thought to be central to the atherosclerotic cascade, the common denominator in the pathophysiology of major adverse cardiovascular and cerebral events. OxLDL acts as an antigen, which is recognised by macrophages and induces foam cell formation, with ensuing plaque lipid core development, apoptosis, cell death, and cytokine production [2]. OxLDL is formed through the post-translational oxidative modification of LDL that has crossed the intimal arterial layer, becoming trapped beneath it in the sub-intimal space. OxLDL is actually a collective term, reflecting a wide variety of oxidative changes to the LDL particle, including aldehyde adduction, such as malondialdehyde (MDA) onto Apolipoprotein B, the principle protein of LDL. Notably, post mortem studies have suggested that lesions with greater oxLDL deposition may be at increased risk of plaque rupture [3,4]. Therefore, oxLDL is an ideal target for the investigation of potential identification of atherosclerotic plaques prone to rupture. Moreover, multiple studies have demonstrated that elevated levels of serum oxLDL are associated with the development of future CVD and often poorer prognosis, with conceivable clinical use as a biomarker [5,6,7,8,9,10,11,12].

Moving on from the measurement of oxLDL, the measurement of autoantibodies to oxLDL may allow improved cardiovascular risk stratification. Auto-antibodies may be generated for any of the oxidation specific epitopes that have been developed for the oxidative modification of LDL. Many studies have been performed to explore the association between autoantibodies to oxLDL and CVD. However, it is somewhat difficult to draw clear conclusions, given the contradictory findings, variable study design with dissimilar endpoints, as well as different laboratory assays and techniques.

This systematic review aims to evaluate the studies that have been performed to assess the association between autoantibodies to oxLDL and cardiovascular mortality. There are autoantibodies to a wide variety of oxidation specific epitopes that have been evaluated in the literature, but this systematic review focuses on MDA-LDL, copper-oxidized LDL (Cu-oxLDL), and oxLDL (with non-specified oxidative-modified epitopes on LDL), the most widely reported autoantibodies. Additionally, given the contrasting endpoints used and the broad clinical spectrum covered by CVD, this review focuses on CAD with cardiac endpoints, including CAD severity as assessed at coronary angiography (CAG).

## 2. Materials and Methods

### 2.1. Search Strategy

In June 2018 we systematically searched Medline, Embase, Web of Science and Google Scholar electronic databases for relevant literature using different variations, abbreviations and language variations of the following keywords: oxidized low-density lipoprotein, antibodies, autoantibodies, atherosclerosis, and coronary artery disease (detailed search strategy given in Supplementary File S1). In addition, we hand-searched the reference lists of relevant reviews in the field for the identification of additional publications for inclusion. Our search was limited to peer-reviewed articles that have been published in English and that carried out studies on human adults. In addition, the studies had to focus on healthy individuals or patients with CAD. Studies that centred primarily on patients with autoimmune diseases, for example Systemic Lupus Erythematosus, were excluded.

### 2.2. Review Method and Selection Criteria

Studies were eligible in the study, either, if they reported an association between IgG and/or IgM autoantibodies to MDA-LDL, Cu-oxLDL, or a total oxLDL and the occurrence of cardiovascular events during a follow-up of at least one year; or if they reported an association between IgG and/or IgM autoantibodies to MDA-LDL, Cu-oxLDL, or total oxLDL with degree of coronary atherosclerosis assessed by coronary angiography or other coronary imaging modality. We excluded cross-sectional studies that focused on differences in serum levels of MDA-LDL, Cu-oxLDL, or total oxLDL autoantibodies between patients with different types of CAD (myocardial infarction (MI), unstable angina, stable angina or prior MI), as the degree of CAD cannot be quantified using these definitions. In addition, studies were excluded that did not specify which subclass of autoantibody were evaluated (i.e., total versus IgG versus IgM), so as to permit analysis per immunoglobulin (Ig) subtype, given the diverging associations with CVD reported in the literature.

Two physician reviewers (VJB and MMV) independently screened the publications for eligibility at title or abstract level. The remaining publications underwent full text review. The differences between reviewers regarding study selection were resolved by a third reviewer (RK). To assess the quality of the included studies, we used the Newcastle-Ottawa Scale for cohort studies and case-control studies. A modified scale was used for cross-sectional studies [13].

### 2.3. Data Extraction

For each included study, data was extracted independently by two reviewers (VJB and MMV). Subsequently, the information was compared and merged, and discrepancies were resolved by consensus. The extracted data consisted of study and patient characteristics, autoantibodies of interest, method for determining the antibody levels, and the association between the antibody levels and the clinical events or degree of CAD, as quantified by imaging. If the studies used uni- and multi-variable models to assess these associations, we chose to include the results of the multivariable model adjusted for the most confounders.

## 3. Results

**Figure 1:**
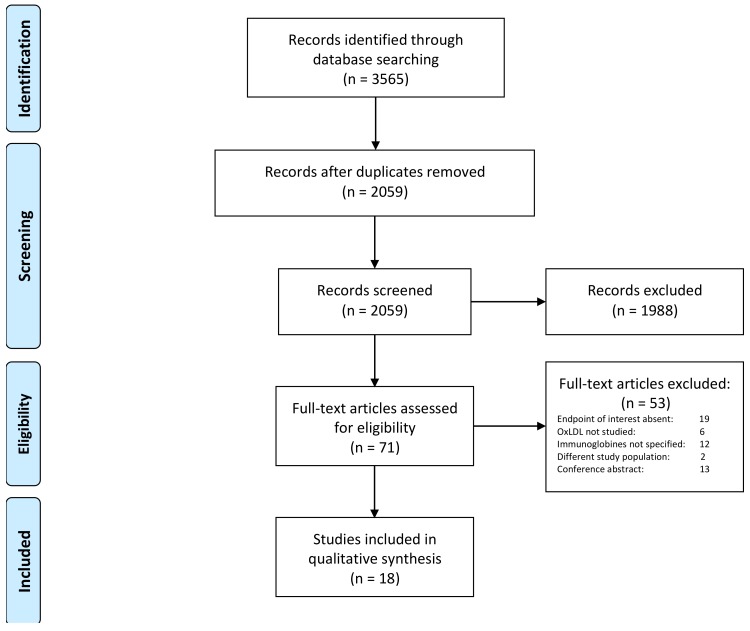
Flow diagram.

The systematic literature search yielded a total of 2059 records, potentially eligible for our current analysis. Based on the titles and abstract, 1988 records were excluded, hence 71 studies underwent full text review. Subsequently, 53 studies were further excluded, as they did not meet the inclusion criteria. 18 original studies were included in the systematic review (Figure 1). The study populations and the baseline characteristics of the included studies are shown in Table 1. The results of the quality assessments for all studies are provided in Table S1.

### 3.1. Autoantibodies against oxLDL and Severity of CAD

A total of 11 cross-sectional studies were identified that explored the association between the degree of CAD, as quantified by CAG, intravascular ultrasound (IVUS) or near-infrared spectroscopy (NIRS) and IgG oxLDL autoantibodies [15,18,22,23,24,25,26,27,28,29,30], whilst five studies evaluated the relationship with IgM oxLDL autoantibodies [18,22,25,26,30]. Nine of these studies investigated all consecutive patients undergoing clinically indicated CAG [15,18,22,23,24,26,28,29,30], one study included only women undergoing CAG [25], and one study included solely patients with ST-segment elevation MI (STEMI) [27]. The endpoints were defined as number of diseased coronary arteries [18,23,26,27,29]; the Gensini score [24]; the Duke score [15] (both being composite scores for CAD lesion location and severity); a custom angiographic severity score [25]; or plaque characteristics determined by IVUS and lipid core burden index (LCBI) measured by NIRS, both measured in a non-culprit vessel [22]. Two studies divided their CAG patients in groups with at least one stenosis >50% and a group without stenosis >50%, and compared these two groups with healthy individuals [28,30]. An overview of the study results is described in Table 2.

Except for Che et al. [24] and Gruzdeva et al. [27], nine studies described a non-significant association between IgG anti-oxLDL antibodies or anti-MDA-LDL antibodies and the severity of CAD, as quantified at CAG [15,18,22,23,25,26,28,29,30]. Che et al. [24] found a negative association between the natural logarithm of the (Gensini-score + 1) and serum IgG anti-oxLDL autoantibodies in 154 consecutive CAG patients. However, their findings seem to be heavily (negatively) biased by an outlying value of IgG and were not confirmed in multivariable analysis. In contrast to Che et al., Gruzdeva et al. [27] reported a positive association between IgG anti-oxLDL antibodies and the number of coronary arteries with a stenosis of >75% in patients undergoing CAG for STEMI. In a univariable logistic regression model, with the dependent variable of one-vessel disease versus multi-vessel disease, IgG autoantibodies to oxLDL had a discriminative ability, expressed by a c-statistic of 0.85.

The association between IgM anti-MDA-LDL or total anti-oxLDL antibodies has been investigated less extensively. In the study by Garrido-Sanchez et al. [26], an inverse relationship between IgM anti-ox-LDL levels and the number of diseased coronary arteries was found (*p* < 0.005). The same association was reported by Tsimikas et al. [18] for both IgM anti-MDA-LDL (*p* = 0.027) and IgM anti-Cu-oxLDL (*p* = 0.030). However, in a multivariable logistic regression model with the presence of obstructive CAD (defined as 1 or more stenosis of >50%) as the dependent variable, IgM anti-oxLDL level was not an independent predictor of obstructive CAD. Similarly, van den Berg et al. [22] reported that plaque burden or volume in a non-culprit vessel, as determined by IVUS measurements, was not significantly associated with IgM anti-oxLDL. In contrast, IgM anti-oxLDL was inversely associated with the degree of necrotic core in the same lesion and with the lipid core burden index (LCBI)-score of the worst 4mm in the measured segment [22]. The study by Chen et al. [25] also revealed that, higher IgM antibodies levels were associated with less severe CAD. In this study, patients with no, to very, mild (<20% stenosis) CAD had significantly higher IgM levels than patients with at least one stenosis of >20%, after adjusting for the effects of age, smoking, total cholesterol, and LDL cholesterol. This inverse relationship seemed to be more profound in Caucasian women than in Afro-American women. However, when IgM anti-oxLDL serum levels were correlated with a custom-made CAD severity score that accounted for severity of stenosis, adjusted for collaterals and lesion location, no significant association was found. Finally, although, the study by Soto et al. [30] did find higher IgM anti-oxLDL antibody levels in healthy controls and patients, without significant CAD, as quantified by CAG than in patients with CAD, these results should be interpreted with caution given only 30 patients were analysed (20 CAG patients and 10 controls).

### 3.2. Autoantibodies against oxLDL and Cardiovascular Events in Patients without Established CAD

We found four cohorts [10,14,17,19] and three nested case-control studies [20,21,22] that assessed the association between IgG and IgM anti-oxLDL and cardiovascular events in subjects without established CAD. There was significant variation in the frequency of cardiovascular risk factors present amongst the population-based studies. For example, Khamis et al. and Van den Berg et al. conducted their studies in subjects with hypertension [20,22]. Study populations generally consisted mainly of Caucasians. Whereas, Prasad et al. included subjects differing in ethnicity (Caucasian, Black and Hispanic) [17]. All seven studies quantified autoantibodies in blood samples collected at baseline and assessed long-term cardiovascular outcomes. Björkbacka et al. additionally distinguished between IgM and IgG autoantibodies against amino acid sequences 661–680 (p45) and 3136–3155 (p210) [14].

All seven studies assessed the association between IgG oxLDL autoantibodies and cardiovascular end points (Table 3). Both Tsimikas et al. and Prasad et al. found that elevated levels of IgG anti-oxLDL were associated with a greater risk of developing future events (hazard ratio (HR) per standard deviation (SD) increase: 1.18, 95% confidence interval (CI) 1.03–1.37, and HR for fourth quartile vs first quartile: 1.97, 95%CI 1.30–2.99, respectively) [10,17]. Conversely, Khamis et al. found a protective association between IgG anti-oxLDL and cardiovascular end points, with cases having lower levels of IgG anti-oxLDL than controls (Odds ratio (OR) for third versus first tertile: 0.74, 95%CI 0.56–0.97) [20]. The remaining four studies that assessed the association between IgG anti-oxLDL levels and cardiovascular end-points did not detect significant associations.

Six studies assessed the association between IgM anti-oxLDL levels and cardiovascular end-points (Table 3). Tsimikas et al. found that higher serum levels of IgM oxLDL autoantibodies were associated with a lower risk of developing cardiovascular end points (HR/SD increase: 0.69, 95%CI 0.50–0.95) [10]. Van den Berg et al. also indicated a strong protective effect of IgM anti-MDA-LDL antibodies on future CAD events (OR of third versus first tertile 0.29 (0.11, 0.76; *p* = 0.012; *p* = 0.016 for trend) [22]. A similar protective association was seen in the study by Björkbacka et al. for IgM-p45 autoantibodies, with an adjusted HR for third vs first tertile of 0.59 (95%CI: 0.46–0.76; *p* < 0.001). No significant association between IgM-p210 autoantibodies and cardiovascular end points was observed [14]. The remaining three studies did not find a significant association between IgM anti-MDA-LDL antibodies and events [17,20,21].

### 3.3. Autoantibodies against oxLDL and Cardiovascular Events in Patients with Established CAD

Only two studies investigated the prognostic value of autoantibodies to oxLDL in CAD patients; one additional study was identified that included patients with known CVD (summarised in Table 4). The two CAD studies included (mainly Caucasian) patients undergoing clinically indicated CAG [15,18], and the CVD study included patients undergoing carotid endarterectomy [16]. Antibody levels, determined from baseline samples and taken prior to intervention, were evaluated with events during follow-up. Among 168 patients undergoing carotid endarterectomy, Meeuwsen et al. [16] did not find any significant differences in baseline IgG and IgM anti-oxLDL in patients by whom the endpoint, a composite of cardiac death, non-fatal MI, stroke, and percutaneous or peripheral intervention, later occurred and in whom it did not occur. Similar to the study by Meeuwsen et al., Tsimikas et al. reported no significant associations between both IgG and IgM anti-MDA-LDL levels of 504 patients included in the BRUNECK study and cardiovascular events during a median follow-up of 4 years. However, during CAG, only 271 patients had obstructive CAD, the number of events (44; 20 all-cause deaths, 14 MIs, and 10 strokes) was low and the paper does not mention the statistical techniques used for analysis. On the contrary, Maiolino et al. [15] reported different results in patients undergoing clinically indicated CAG. In blood samples of 544 patients from the GENICA (Genetic and ENvironmental factors in Coronary Atherosclerosis) study, there was a significant positive association between IgG anti-MDA-LDL levels and both, the occurrence of cardiovascular death (*p* = 0.04), and the occurrence of cardiovascular events (*p* = 0.005). In an additional analysis, 136 of the 140 patients from the highest IgG anti-MDA-LDL quartile were successfully matched to 136 patients from the other three quartiles, based on a propensity score that was computed with the most well-identified clinical characteristics associated with cardiovascular events. When comparing patients with high oxLDL antibody levels with their propensity matched controls, the patients with high levels had significantly less chance of cardiovascular death-free survival (83.1% vs 89.0%, *p* = 0.025) and less chance of cardiovascular event-free survival (69.2% versus 77.7%, *p* = 0.030), during a median follow-up of 7.2 years.

## 4. Discussion

This systematic review highlights that the results on associations between anti-oxLDL antibodies and cardiovascular endpoints, described in the literature so far, are heterogeneous and several aspects remain inconclusive. Moreover, despite a comprehensive search methodology, only 18 original studies were identified that satisfied the broad inclusion/exclusion criteria. The studies identified were generally of a very high standard, as assessed by the (modified) Newcastle-Ottawa Scale, except for low scores reported for some of the cross-sectional studies (Supplementary Table S1). All of the included studies reported on IgG anti-oxLDL antibodies, with a smaller proportion reporting associations with IgM anti-oxLDL antibodies.

The studies investigating the relationship between anti-oxLDL antibodies and CAD as quantified by CAG (Table 2) varied in inclusion criteria and endpoint definition, and were often characterized by methodologically weak designs (Table S1). Given the currently available evidence, there is little to support the hypothesis that IgG anti-oxLDL antibodies and CAD severity are related, except for perhaps in STEMI patients. IgM antibodies against oxLDL seem to have an inverse relationship with the CAD severity. However, it is unclear if this relationship is also as strongly apparent when adjusted for confounders. It is important to note that all the studies evaluating IgM also assessed IgG anti-oxLDL antibodies, yet no relationship is found with IgG. Conversely, a clear association is found with IgM anti-oxLDL antibodies in the same study populations. This further strengthens the lack of an identified relationship between IgG anti-oxLDL antibodies and CAD.

The inverse relationship between IgM antibodies and the necrotic core volume/ LCBI score, as demonstrated by van den Berg et al. [22], is provoking and promising, as both endpoints have been shown previously to correlate with future cardiovascular events [31]. Future studies should use a well-validated score for CAD severity, use a regression analysis to establish the relationship between this (semi-) continuous endpoint and antibodies, as well as perform multivariable adjustments in order to confirm this relationship. In addition, there should be sufficient patients included to perform sub-group analysis, based on CAG indication (e.g., STEMI, non-STEMI, stable CAD).

The seven studies (Table 3) conducted to assess the association of anti-oxLDL antibodies with cardiovascular endpoints in patients without objectified CAD, demonstrated inconclusive conclusions with IgG anti-oxLDL antibody levels. Conversely, it seems that IgM anti-oxLDL levels are inversely associated with the events. Moreover, the divergent results of the studies investigating cardiovascular endpoints with anti-oxLDL antibodies, in patients undergoing clinically indicated CAG (Table 4), may be partially explained by differences in design, statistical analysis, and number of events occurring. Current studies reported mixed endpoints, vastly different endpoints between studies, and the use of different experimental techniques or antibodies. Well-designed prospective studies, with well-characterised populations, amongst diagnosed CAD patients or at-risk populations, will be needed for further investigations if anti-oxLDL antibody levels are indeed associated with cardiovascular events. It may also be prudent to focus on coronary heart disease endpoints (i.e., non-fatal MI or cardiac death) as these are objective endpoints with more diagnostic certainty than their vascular or cerebrovascular counterparts. Naturally, this will require greater patient recruitment due to lower event rates; but may serve to more confidently assess potential associations. Another confounder to consider is the interaction of cardiovascular preventative medications. Statins have been demonstrated to somewhat counter-intuitively reduce IgM and increase IgG anti-MDA-LDL antibody levels, independent of dose [32]. Whereas, in the SARD (Standard versus high-dose therApy with Rosuvastatin for lipiD lowering) randomised clinical trial, rosuvastatin reduced total oxLDL levels in a dose-dependent manner. Thus, the interaction of statins with oxLDL is also complex and requires further clarification.

Despite the differences in study design and the presence of confounders discussed above, it appears that IgM anti-oxLDL antibodies indicate protection from CV events and more severe CAD, whilst the relationship with IgG is more complex and difficult to elucidate. The biological role of these autoantibodies needs to be considered, more than just in their putative role as biomarkers in clinical practice. It is hypothesised that, as a key component of the innate immune system, IgM anti-oxLDL antibodies perform homeostatic functions, maintaining the equilibrium of atherosclerosis development. Perhaps in the presence of overwhelming stimuli, such as traditional cardiovascular risk factors or other inflammatory triggers, a mal-adaptive immune response occurs, with immunoglobulin class switching to IgG and accelerated atheroma deposition/plaque rupture [2]. Interestingly, a recent set of experiments using a single-chain variable fragment of E06, a naturally occurring IgM antibody that inhibits the uptake of oxLDL into macrophages, has demonstrated reduced levels of atheroma development, systemic inflammation, and even prolonged life in LDL-receptor-null mice fed a high-cholesterol diet [33]. Thus, this study provides plausible mechanistic evidence for the theorised beneficial anti-inflammatory and anti-atherosclerotic actions of IgM anti-oxLDL antibodies.

### Limitations

Our systematic review was mainly limited by the difficulty of comparing between studies in this particular research field. This is due to several reasons. Firstly, laboratories are currently each using their own methods to measure and quantify the various anti- OxLDL antibody levels, since assays that measure these levels have not yet been standardised or commercialised. As a result, although we used broad inclusion criteria, anti-OxLDL antibody levels that were reported in the 18 included studies, could not be directly compared. Secondly, although we focused on healthy populations or patient with CVD, and excluded patients with additional diseases, such as auto-immune diseases, residual heterogeneity between the patient populations of the various included studies remain. Finally, most included studies used (unstandardized) composite endpoints but failed to report the results for each individual endpoint.

## 5. Conclusions

Despite the paucity of studies and lack of conclusive data in the literature, this systematic review has highlighted clear signals of association between anti-oxLDL antibodies and CAD. IgM anti-oxLDL antibodies appear to indicate protection from more severe CAD and possibly cardiovascular events, whilst the relationship with IgG is more complex and difficult to elucidate. Further studies, with well-characterised prospective cohorts, will be important to clarify these associations.

## Figures and Tables

**Table 1:** Baseline characteristics of the included studies.

Author	Year	Population (n)	Investigated Biomarkers	Sample Size		Age in Years Mean (SD)		Male Gender (%)		DM (%)		HTN (%)		Current Smoking (%)	
				CAD	no CAD	CAD	no CAD	CAD	no CAD	CAD	no CAD	CAD	no CAD	CAD	no CAD
Cohort studies															
Björkbacka [14]	2016	Population-based prospective cohort (5393)	anti-p45 IgG, anti-p45 IgM, anti-p210 IgG, anti-p210 IgM	398	4995	61.1 [57.2–64.7] *	57.6 [52.2–62.5] *	61.6	39.8	19.8	7.4	81.2	62.5	33.8	26.1
Maiolino [15]	2012	CAG patients (733) b	IgG anti-MDA-LDL	733		63.3		78.4		NR		NR		7.3	
Meeuwsen [16]	2017	168 endarterectomy patients	IgG anti-oxLDL, IgM anti-oxLDL	168		70.1 (9.6)		62.8		23.8		86.6		35.7	
Prasad [17]	2017	Population-based prospective cohort (3509)	IgG anti-MDA-LDL, IgM anti-MDA-LDL	190	2914	43.7 (10.1) †		44.1†		11.6†		34.4†		29.3†	
Tsimikas [18]	2007	CAG patients (504) a	IgG anti-MDA-LDL, IgM anti-MDA-LDL	504		60.1		61.7		NR		46.0		7.9	
Tsimikas [10]	2012	Population-based prospective cohort (765)	IgG anti-cu-oxLDL, IgM anti-MDA-LDL	138	627	68.8 (10.5)	61.4 (10.9)	48.5	59.4	13.8	6.9	75.4	66.0	17.4	20.4
Wilson [19]	2006	Population-based prospective cohort (2619)	IgG anti-MDA-LDL	151	2468	49.52 †		NR	NR	NR	NR	NR	NR	NR	NR
Case-control studies															
Khamis^c^ [20]	2016	Hypertensive patients receiving blood pressure-lowering treatment (1852)	IgG anti-MDA-LDL, IgM anti-MDA-LDL	485	1367	65.3 (7.8)	65.3 (7.6)	84.5	84.9	30.9	26.3	NR	NR	7.8	7.6
Ravandi [21]	2011	Population-based prospective cohort (2471)	IgG anti-MDA-LDL, IgM anti-MDA-LDL	748	1723	65.4 (7.8)	65.4 (7.8)	62.8	61.6	NR	NR	NR	NR	15.5	8.6
van den Berg ^d^ [22]	2018	87 subjects with CHD, 227 subjects free of CHD	IgG anti-MDA-LDL, IgM anti-MDA-LDL	87	227	60.4 (6.3)	59.8 (6.4)	67.8	64.8	14.9	7.9	100	100	8	6.6
Cross-sectional studies															
Bilgen [23]	2005	CAD patients (136). healthy controls (31)	IgG anti-oxLDL	136	31	57.6 (11.3)	53.5 (10.2)	70.5	67.8	13.2	0	30.9	0	47.8	0
Che [24]	2011	CAG patients (154)	IgG anti-oxLDL	117	37	63.7 (10.6)	62.0 (11.5)	63.4	64.9	24.8	16.2	64.1	70.3	43.6	24.3
Chen [25]	2011	CAG patients (558)	IgG anti-MDA-LDL, IgM anti-MDA-LDL	334	224	60.7	53.2	0	0	31.4	17.9	63.2	47.3	21.3	16.5
Garrido-Sanchez [26]	2009	CAG patients (236)	IgG anti-oxLDL, IgM anti-oxLDL	NR	NR	NR	NR	NR	NR	NR	NR	NR	NR	NR	NR
Gruzdeva [27]	2014	STEMI patients (400), 33 healthy controls	IgG anti-oxLDL	400	NR	60.3 (1.1)	NR	67.5	NR	NR	NR	69.8	NR	42	NR
Maiolino [15]	2012	CAG patients (733) b	IgG anti-MDA-LDL	733		63.3		78.4		NR		NR		7.3	
Moohebati [28]	2013	CAG patients (63), healthy controls (24)	IgG anti-oxLDL	31	56	59.4 (10.1)	58.3	38.7	58.9	41.9	10.7	64.5	44.6	51.6	25
Rossi [29]	2003	CAG patients (529)	IgG anti-MDA-LDL	445	84	63	62	NR	NR	NR	NR	NR	NR	NR	NR
Soto [30]	2009	CAG patients (20), healthy controls (10)	IgG anti-oxLDL, IgM anti-oxLDL	13	17	59	36.5	100	52.9	23.1	0	69.2	11.8	46.2	11.8
Tsimikas [18]	2007	CAG patients (504) a	IgG anti-MDA-LDL, IgM anti-MDA-LDL	504		60.1		61.7		NR		46.0		7.9	
van den Berg ^d^ [22]	2018	87 subjects with CHD, 227 subjects free of CHD	IgG anti-MDA-LDL, IgM anti-MDA-LDL	87	227	60.4 (6.3)	59.8 (6.4)	67.8	64.8	14.9	7.9	100	100	8	6.6

If CAG patients were classified as having normal coronary arteries, their baseline characteristics are mentioned under ‘without CAD’ and combined with healthy controls, if needed. Note that experimental biomarker assays are methodologically heterogenous. Please refer to the original publications for assay details. a: all patients were divided into either 2 groups; one with at least one stenosis with a diameter > 50% and one group without. As for the latter group, we cannot determine the baseline characteristics for the patients without any CAD. We chose to summarize all the baseline characteristics under known CAD. b: of which 570 used for analysis. c: Case-control constructed from participants of a randomized controlled trial comparing two blood pressure lowering regimes: (1) a beta-blocker with a thiazide diuretic and a (2) dihydropyridine calcium channel blocker with an angiotensin-converting enzyme inhibitor. d: two separate cohorts are discussed in this paper, both are included separately in the systematic review. * median [IQR]. † Baseline characteristics only available for the entire group. CAD: coronary artery disease, CAG: coronary angiography, DM: diabetes mellitus, HTN: hypertension, MI: myocardial infarction, NR: not reported; SD: standard deviation.

**Table 2:** Relationship between anti-oxidized low density lipoprotein (anti-oxLDL) antibodies and coronary artery disease assessed by coronary imaging.

Author	Index Group (*n*)	Reference Group (*n*)	Statistical Method	Confounders Adjusted for in Statistical Analysis	Outcome	IgG Anti-oxLDL	IgM Anti-oxLDL
Bilgen [23]	CAG patients (136)	Healthy controls (36)	ANOVA with post-hoc Tukey		Diseased coronary arteries (*n*)	NS	
Che [24]	CAG patients (154)		Multiple linear stepwise regression	hs-CRP, fasting glucose, serum albumin	ln(Gensini score + 1)	Beta −0.020 (−0.033, −0.006)	
Chen [25]	Female CAG patients (558)		ANOVA	Age, smoking, and total and LDL cholesterol	<20% stenosis vs >20% stenosis	NS	↓
Garrido-Sanchez [26]	CAG patiens (236)		NR		Diseased coronary arteries (*n*)	NS	↓
Gruzdeva [27]	STEMI patients (400)	Healthy controls (33)	Kruskal-Wallis, followed by Mann-Whitney with Bonferonni correction		Diseased coronary arteries (>75%) (*n*)	↑	
Maiolino [15]	CAG patients (733)		ANOVA		Duke CAD score	NS	
Moohebati [28]	CAG patients (63)	Healthy controls (24)	ANOVA		1 or more stenosis (>50%) vs no stenosis vs healthy control	NS	
Rossi [29]	CAG patients (529)		ANOVA		Diseased coronary arteries (>50%) (*n*)	NS	
Soto [30]	CAG patients (20)	Healthy controls (10)	ANOVA		1 or more stenosis (>50%) vs no stenosis vs healthy control	unclear	↓
Tsimikas [18]	CAG patients (504)		Logistic regression	Age, gender, LDL-C, smoking, HDL, hypertension	obstructive CAD (>50%) vs no obstructive CAD	NS, OR not given	NS, OR not given
van den Berg [22]	CAG patients (143)		Linear regression, trend test among four quartiles	Age, gender, diabetes, smoking, previous statin use, LDL and HDL cholesterol	IVUS determined plaque characteristics in a non-culprit vessel, NIRS determined LCBI score in a non-culprit vessel	NS	↓

↑ Indicates a significantly positive association with outcome (high Ig-levels are associated with more CAD), ↓ Indicates a significantly inverse association with outcome (high Ig-levels are associated with less CAD). CAD: coronary artery disease, CAG: coronary angiography, hs-CRP: high-sensitive C-reactive protein, Ig: immunoglobulin, Ig: immunoglobulin, NR: not reported, NS: Not significant, OxLDL: oxidized LDL, STEMI: ST-elevation myocardial infarction.

**Table 3:** Anti-oxLDL antibodies and cardiac endpoints in subjects without prevalent coronary artery disease.

Author	Study	Follow-up Period, Years	Statistical Method	Matching Variables	Confounders Adjusted for in Statistical Analysis	Endpoint	IgG Anti-oxLDL	IgM Anti-oxLDL
Björkbacka [14]	Cohort study	>15	Multivariable Cox regression		Age, gender, LDL, HDL, SBP, triglycerides, hs-CRP, smoking, anti-hypertensive treatment, diabetes	Fatal and non-fatal MI, ischemic heart disease	Adjusted HR between tertiles: IgG anti-MDA-LDL p45: 1.00 vs 0.85 (0.66–1.11) vs 0.89 (0.68–1.16) p trend:0.89; IgG anti-MDA-LDL p210: 1.00 vs 0.81 (0.63–1.06) vs 0.96 (0.74–1.23) p trend: 0.29	Adjusted HR between tertiles: IgM anti-MDA-LDL p45: 1.00 vs 0.79 (0.t62–0.99) vs 0.59 (0.46–0.76) p trend: 0.001; IgM anti-MDA-LDL p210: 1.00 vs 0.81 (0.63–1.06) vs 0.96 (0.74–1.23) p trend: 0.29
Khamis [20]	Nested case-control study	5.5 (median)	Conditional logistic regression	Age, Gender	Smoking, diabetes, SBP, total cholesterol, HDL, creatinine, BMI, family history of CAD, anti-hypertensive and statin treatment, CRP, NTproBNP	Fatal coronary heart disease, symptomatic non-fatal MI, coronary revascularization, fatal and non-fatal stroke	Adjusted OR per SD increase: IgG anti-MDA-LDL 0.83 (0.72–0.95)	Adjusted OR per SD increase: IgM anti-MDA-LDL 0.90 (0.78–1.03)
Prasad [17]	Cohort study	10.5 (median)	Multivariabel Cox regression		Age, gender, hypertension, diabetes, smoking, BMI, LDL, HDL, triglycerides, ethnicity	Cardiac death, non-fatal MI, stroke/TIA, unstable angina requiring hospitalization and arterial vascularization that included CABG, PCI, carotid endarterectomy, carotid stenting and peripheral artery revascularization	Adjusted HR 4th quartile vs first quartile: IgG anti-MDA-LDL: 1.97 (1.30–2.99)	Adjusted HR 4th quartile vs first quartile: IgM anti-MDA-LDL: 0.96 (0.63–1.45)
Ravandi [21]	Nested case-control study	6 (mean)	Conditional logistic regression	Age, gender, time of enrollment	Diabetes, smoking, SBP, LDL, HDL	cardiac death, hospital admission with CAD	Adjusted OR between tertiles: IgG anti-MDA-LDL 1.00 vs 0.80 (0.64–1.00) vs 0.94 (0.75–1.18), ptrend: 0.4	Adjusted OR between tertiles: IgM anti-MDA-LDL: 1.00 vs 1.01 (0.81–1.26) vs 0.91 (0.72–1.15), p trend: 0.6
Tsimikas [10]	Cohort study	> 15	Multivariabel Cox regression		Age, Gender, previous CVD, SBP, smoking, diabetes, ferritin, LDL, HDL, alcohol consumption, social status, sport activity, CRP	Stroke, MI, new-onset unstable angina, acute coronary interventions and cardiac death	Adjusted HR: IgG anti-cu-oxLDL: 1.18 (1.03–1.37)	Adjusted HR: IgM anti-MDA-LDL: 0.69 (0.50–0.95)
Van den Berg [22]	Nested case-control study	4.5 (mean)	Conditional logistic regression	Age, gender, time of enrollment	Smoking, diabetes, baseline HDL, blood pressure treatments, either total IgG or total IgM.	fatal MI, non-fatal MI Q-wave criterium, non-fatal MI T-wave criterium, sudden death, new-onset ischemic heart disease or new-onset congestive heart failure	Adjusted OR between tertiles per SD increase in loge IgG anti-MDA-LDL: 1.00 vs 0.65(0.33–1.28) vs 0.93 (0.45–1.92) ptrend: 0.82	Adjusted OR between tertiles per SD increase in loge IgM anti-MDA-LDL: 1.00 vs 0.90 (0.45–1.80) vs 0.29 (0.11–0.76) ptrend: 0.016
Wilson [19]	Cohort study	> 8	Multivariabel Cox regression		Age, total cholesterol, HDL, smoking, SBP	Angina pectoris, unstable anginga pectoris, MI, cardiac death, TIA and stroke	Adjusted HR males and females per 1000 units: IgG anti-MDA-LDL: 1.00 (1-1); IgG anti-MDA-LDL: 1.00 (1-1)	

**Legend:** HR: hazard ratio, Ig: immunoglobulin, LDL: low density lipoprotein, MDA: malondiadehyde, OR: odds ratio, SD: standard deviation.

**Table 4:** Anti-oxLDL antibodies and cardiac endpoints in subjects undergoing clinically indicated coronary angiography (CAG).

Author	Index Group (n)	Reference Group (n)	Follow-up Period, Years	Statistical Method	Matching Variables	Confounders Adjusted for in Statistical Analysis	Outcome	IgG Anti-oxLDL	IgM Anti-oxLDL
Maiolino [15]	CAG patients from highest IgG anti-MDA-LDL quartile (136)	CAG patients from the lower three IgG anti-MDA-LDL quartiles matched based on propensity score	7.2 (median)	Kaplan-Meier	Gender, Age, BMI, LDL- and HDL-cholesterol, triglycerides, serum creatinine, homocysteine, glycemia, serum sodium concentration, heart rate arterial hypertension, smoking habit, LVEF, the Duke Prognostic Index of coronary athersosclerotic burden, length of follow-up, history and treatment variables		Cardiac death, composite of non-fatal MI, non-fatal stroke, and cardiac death	Event-free survival; 1-3rd Q vs. 4th Q: 77.7% vs. 69.2%	
Meeuwsen [16]	Carotid endarterectomy patients (168)		3	Cox regression			Composite of cardiac death, stroke, non-fatal MI, coronary intervention, and peripheral intervention (including amputation)	HR 1.01 (0.98–1.03)	
Tsimikas [18]	CAG patients (504)		4 (median)	NR			Composite of non-fatal MI, non-fatal stroke, and cardiac death	NS	NS

↑ Indicates a significantly higher level in the index group compared with the reference group, ↓ Indicates a significantly higher level in the index group compared with the reference group. BMI: body mass index, CAG: coronary angiography, HDL: high density lipoprotein, Ig: immunoglobulin, LDL: low density lipoprotein, LVEF: left ventricular ejection fraction, NR: not reported, NS: not significant, MDA: malondiadehyde, MI: myocardial infarction, oxLDL: oxidized LDL.

## Supplementary Materials

The following are available online at https://www.mdpi.com/2076-3921/8/10/484/s1, File S1: Search strategy, and Table S1: Quality control.

## References

[1] WHO (2018). The Top 10 Causes of Death.

[2] Hartley A., Haskard D., Khamis R. (2019). Oxidized ldl and anti-oxidized ldl antibodies in atherosclerosis–novel insights and future directions in diagnosis and therapy. Trends Cardiovasc. Med..

[3] Fefer P., Tsimikas S., Segev A., Sparkes J., Otsuma F., Kolodgie F., Virmani R., Juliano J., Charron T., Strauss B.H. (2012). The role of oxidized phospholipids, lipoprotein (a) and biomarkers of oxidized lipoproteins in chronically occluded coronary arteries in sudden cardiac death and following successful percutaneous revascularization. Cardiovasc. Revascularization Med..

[4] Van Dijk R.A., Kolodgie F., Ravandi A., Leibundgut G., Hu P.P., Prasad A., Mahmud E., Dennis E., Curtiss L.K., Witztum J.L. (2012). Differential expression of oxidation-specific epitopes and apolipoprotein (a) in progressing and ruptured human coronary and carotid atherosclerotic lesions. J. Lipid Res..

[5] Bertoia M.L., Pai J.K., Lee J.-H., Taleb A., Joosten M.M., Mittleman M.A., Yang X., Witztum J.L., Rimm E.B., Tsimikas S. (2013). Oxidation-specific biomarkers and risk of peripheral artery disease. J. Am. Coll. Cardiol..

[6] Byun Y.S., Yang X., Bao W., DeMicco D., Laskey R., Witztum J.L., Tsimikas S., Investigators S.T. (2017). Oxidized phospholipids on apolipoprotein b-100 and recurrent ischemic events following stroke or transient ischemic attack. J. Am. Coll. Cardiol..

[7] Gao S., Zhao D., Wang M., Zhao F., Han X., Qi Y., Liu J. (2017). Association between circulating oxidized ldl and atherosclerotic cardiovascular disease: A meta-analysis of observational studies. Can. J. Cardiol..

[8] Holvoet P., Vanhaecke J., Janssens S., Van de Werf F., Collen D. (1998). Oxidized ldl and malondialdehyde-modified ldl in patients with acute coronary syndromes and stable coronary artery disease. Circulation.

[9] Nishi K., Itabe H., Uno M., Kitazato K.T., Horiguchi H., Shinno K., Nagahiro S. (2002). Oxidized ldl in carotid plaques and plasma associates with plaque instability. Arterioscler. Thromb. Vasc. Biol..

[10] Tsimikas S., Willeit P., Willeit J., Santer P., Mayr M., Xu Q., Mayr A., Witztum J.L., Kiechl S. (2012). Oxidation-specific biomarkers, prospective 15-year cardiovascular and stroke outcomes, and net reclassification of cardiovascular events. J. Am. Coll. Cardiol..

[11] Uno M., Harada M., Takimoto O., Kitazato K.T., Suzue A., Yoneda K., Morita N., Itabe H., Nagahiro S. (2005). Elevation of plasma oxidized ldl in acute stroke patients is associated with ischemic lesions depicted by dwi and predictive of infarct enlargement. Neurol. Res..

[12] Wang A., Yang Y., Su Z., Yue W., Hao H., Ren L., Wang Y., Cao Y., Wang Y. (2017). Association of oxidized low-density lipoprotein with prognosis of stroke and stroke subtypes. Stroke.

[13] Stang A. (2010). Critical evaluation of the newcastle-ottawa scale for the assessment of the quality of nonrandomized studies in meta-analyses. Eur. J. Epidemiol..

[14] Björkbacka H., Alm R., Persson M., Hedblad B., Nilsson J., Fredrikson G.N. (2016). Low levels of apolipoprotein b-100 autoantibodies are associated with increased risk of coronary events. Arterioscler. Thromb. Vasc. Biol..

[15] Maiolino G., Pedon L., Cesari M., Frigo A.C., Barisa M., Rossitto G., Seccia T.M., Zanchetta M., Rossi G.P. (2013). Antibodies to malondialdehyde oxidized low-density lipoproteins predict long term cardiovascular mortality in high risk patients. Int. J. Cardiol..

[16] Meeuwsen J.A.L., Van Duijvenvoorde A., Gohar A., Kozma M.O., Van de Weg S.M., Gijsberts C.M., Haitjema S., Björkbacka H., Fredrikson G.N., De Borst G.J. (2017). High levels of (un) switched memory b cells are associated with better outcome in patients with advanced atherosclerotic disease. J. Am. Heart Assoc..

[17] Prasad A., Clopton P., Ayers C., Khera A., De Lemos J.A., Witztum J.L., Tsimikas S. (2017). Relationship of autoantibodies to mda-ldl and apob-immune complexes to sex, ethnicity, subclinical atherosclerosis, and cardiovascular events. Arterioscler. Thromb. Vasc. Biol..

[18] Tsimikas S., Brilakis E.S., Lennon R.J., Miller E.R., Witztum J.L., McConnell J.P., Kornman K.S., Berger P.B. (2007). Relationship of igg and igm autoantibodies to oxidized low density lipoprotein with coronary artery disease and cardiovascular events. J. Lipid Res..

[19] Wilson P.W.F., Ben-Yehuda O., McNamara J., Massaro J., Witztum J., Reaven P.D. (2006). Autoantibodies to oxidized ldl and cardiovascular risk: The framingham offspring study. Atherosclerosis.

[20] Khamis R.Y., Hughes A.D., Caga-Anan M., Chang C.L., Boyle J.J., Kojima C., Welsh P., Sattar N., Johns M., Sever P. (2016). High serum immunoglobulin g and m levels predict freedom from adverse cardiovascular events in hypertension: A nested case-control substudy of the anglo-scandinavian cardiac outcomes trial. Ebiomedicine.

[21] Ravandi A., Boekholdt S.M., Mallat Z., Talmud P.J., Kastelein J.J.P., Wareham N.J., Miller E.R., Benessiano J., Tedgui A., Witztum J.L. (2011). Relationship of igg and igm autoantibodies and immune complexes to oxidized ldl with markers of oxidation and inflammation and cardiovascular events: Results from the epic-norfolk study. J. Lipid Res..

[22] Van den Berg V.J., Haskard D.O., Fedorowski A., Hartley A., Kardys I., Caga-Anan M., Akkerhuis K.M., Oemrawsingh R.M., Van Geuns R.J., De Jaegere P. (2018). Igm anti-malondialdehyde low density lipoprotein antibody levels indicate coronary heart disease and necrotic core characteristics in the nordic diltiazem (nordil) study and the integrated imaging and biomarker study 3 (ibis-3). Ebiomedicine.

[23] Bilgen D., Sönmez H., Ekmekçi H., Ulutin T., Öztürk Z., Kökoğlu E., Bayram Ç., Soner A., Domaniç N. (2005). The relationship of tfpi, lp (a), and oxidized ldl antibody levels in patients with coronary artery disease. Clin. Biochem..

[24] Che J., Li G., Wang W., Li Q., Liu H., Chen K., Liu T. (2011). Serum autoantibodies against human oxidized low-density lipoproteins are inversely associated with severity of coronary stenotic lesions calculated by gensini score. Cardiol. J..

[25] Chen Q., Reis S.E., Kammerer C., Craig W., McNamara D.M., Holubkov R., Sharaf B.L., Sopko G., Pauly D.F., Merz C.N.B. (2011). Association of anti-oxidized ldl and candidate genes with severity of coronary stenosis in the women’s ischemia syndrome evaluation study. J. Lipid Res..

[26] Garrido-Sanchez L., Chinchurreta P., Garcia-Fuentes E., Mora M., Tinahones F.J. (2010). A higher level of igm anti-oxidized ldl antibodies is associated with a lower severity of coronary atherosclerosis in patients on statins. Int. J. Cardiol..

[27] Gruzdeva O., Uchasova E., Dyleva Y., Belik E., Karetnikova V., Shilov A., Barbarash O. (2014). Multivessel coronary artery disease, free fatty acids, oxidized ldl and its antibody in myocardial infarction. Lipids Health Dis..

[28] Moohebati M., Kabirirad V., Ghayour-Mobarhan M., Esmaily H., Tavallaie S., Akhavan Rezayat A., Pourghadamyari H., Sahebkar A. (2014). Investigation of serum oxidized low-density lipoprotein igg levels in patients with angiographically defined coronary artery disease. Int. J. Vasc. Med..

[29] Rossi G.P., Cesari M., De Toni R., Zanchetta M., Maiolino G., Pedon L., Ganzaroli C., Maiolino P., Pessina A.C. (2003). Antibodies to oxidized low-density lipoproteins and angiographically assessed coronary artery disease in white patients. Circulation.

[30] Soto Y., Conde H., Aroche R., Brito V., Luaces P., Nasiff A., Obregón Á., Vázquez López A.M. (2009). Autoantibodies to oxidized low density lipoprotein in relation with coronary artery disease. Hum. Antibodies.

[31] Cheng J.M., Garcia-Garcia H.M., De Boer S.P.M., Kardys I., Heo J.H., Akkerhuis K.M., Oemrawsingh R.M., Van Domburg R.T., Ligthart J., Witberg K.T. (2013). In vivo detection of high-risk coronary plaques by radiofrequency intravascular ultrasound and cardiovascular outcome: Results of the atheroremo-ivus study. Eur. Heart J..

[32] Resch U., Tatzber F., Budinsky A., Sinzinger H. (2006). Reduction of oxidative stress and modulation of autoantibodies against modified low-density lipoprotein after rosuvastatin therapy. Br. J. Clin. Pharmacol..

[33] Que X., Hung M.-Y., Yeang C., Gonen A., Prohaska T.A., Sun X., Diehl C., Määttä A., Gaddis D.E., Bowden K. (2018). Oxidized phospholipids are proinflammatory and proatherogenic in hypercholesterolaemic mice. Nature.

