# Supplementary material for: Anti-Oxidized LDL Antibodies and Coronary Artery Disease: A Systematic Review
Source: Antioxidants (Basel). 2019 Oct 15;8(10):484. doi: 10.3390/antiox8100484 (PMC6826549; doi:10.3390/antiox8100484)
Supplement: Supplementary file 1 [file antioxidants-08-00484-s001.pdf]

Supplemental File 1: Search strategy

Database	# of refs	# of refs after de-duplication
Medline Ovid	867	193
embase.com	1365	1343
Web of science	1133	405
Google scholar	200	118
Total	3565	2059

12-06-2018

Medline Ovid
((oxidized low density lipoprotein.mp. AND (Antibodies/ OR Autoantibodies/ OR exp immunoglobulins/ OR exp Mast Cells/)) OR (((oxid* OR ox) ADJ6 (low-density-lipoprotein* OR ldl OR specific-epitope*) ADJ6 (antibod* OR anti OR autoantibod* OR Immune-respon* OR immunoglobulin* OR Igg* OR ige* OR igm* OR iga* OR mast-cell*)) OR ((oxLDL* OR ose) ADJ3 (antibod* OR anti OR autoantibod* OR Immune-respon* OR immunoglobulin* OR Igg* OR ige* OR igm* OR iga* OR mast-cell*))).ab,ti.) AND (exp arteriosclerosis/ OR (atheroscler* OR arterioscler* OR athero-sclero* OR arterio-sclero* OR atherogenes* OR plaque* OR ((peripheral* OR coronar*) ADJ3 artery ADJ3 disease*) OR pad OR poad).ab,ti.) NOT (exp animals/ NOT humans/) AND english.la.

embase.com
('oxidized low density lipoprotein antibody'/exp OR (('oxidized low density lipoprotein'/de OR ('low density lipoprotein'/de AND (oxidation/de OR 'lipid oxidation'/de OR 'lipid peroxidation'/de))) AND ('immune response'/de OR antibody/de OR autoantibody/de OR 'antibody titer'/de OR 'antibody detection'/de OR 'immunoglobulin'/exp OR 'immunoglobulin antibody'/exp OR 'mast cell'/de)) OR (((oxid* OR ox) NEAR/6 (low-density-lipoprotein* OR ldl OR specific-epitope*) NEAR/6 (antibod* OR anti OR autoantibod* OR Immune-respon* OR immunoglobulin* OR Igg* OR ige* OR igm* OR iga* OR mast-cell*)) OR ((oxLDL* OR ose) NEAR/3 (antibod* OR anti OR autoantibod* OR Immune-respon* OR immunoglobulin* OR Igg* OR ige* OR igm* OR iga* OR mast-cell*))):ab,ti) AND ('atherosclerosis'/exp OR 'arteriosclerosis'/exp OR 'peripheral occlusive artery disease'/exp OR 'coronary artery disease'/exp OR (atheroscler* OR arterioscler* OR athero-sclero* OR arterio-sclero* OR atherogenes* OR plaque* OR ((peripheral* OR coronar*) NEAR/3 artery NEAR/3 disease*) OR pad OR poad):ab,ti) NOT ([animals]/lim NOT [humans]/lim) AND [english]/lim

Web of science
TS=(((((oxid* OR ox) NEAR/5 (low-density-lipoprotein* OR ldl OR specific-epitope*) NEAR/5 (antibod* OR anti OR autoantibod* OR Immune-respon* OR immunoglobulin* OR Igg* OR ige* OR igm* OR iga* OR mast-cell*)) OR ((oxLDL* OR ose) NEAR/2 (antibod* OR anti OR

autoantibod* OR Immune-respon* OR immunoglobulin* OR Igg* OR ige* OR igm* OR iga* OR mast-cell*)))) AND ((atherosclero* OR arteriosclero* OR athero-sclero* OR arterio-sclero* OR atherogenes* OR plaque* OR ((peripheral* OR coronar*) NEAR/2 artery NEAR/2 disease*) OR pad OR poad))) AND LA=(english)

Google scholar
"oxidized low-density-lipoprotein"|"ox ldl"|oxldl anti|antibodies|antibody atherosclerosis|arteriosclerosis|atherogenesis|plaque|"peripheral|coronary artery diseases|disease"

Supplemental Table 1. Quality control

	First author	Year	Selection				Comparability*	Outcome/Exposure			Total
cohort studies			*Representativeness of the exposed cohort*	*Selection of the non-exposed cohort*	*Ascertainment of exposure*	*Demonstration that outcome of interest was not present at start of study*	*Comparability of cohorts on the basis of the design or analysis*	*Assessment of outcome*	*Was follow-up long enough for outcomes to occur*	*adequacy of follow-up of cohorts*	number of stars awarded/ maximum number of stars
	Björkbacka	2016	*	NA	*		**	*	*	*	7/8
	Maiolino†	2013	*	NA	*	*	**	*	*		7/8
	Meeuwsen	2017	*	NA	*		**	*	*	*	7/8
	Prasad	2017	*	NA	*		**		*	*	6/8
	Tsimikas†	2007	*	NA	*	*			*		4/8
	Tsimikas	2012	*	NA	*		**	*	*	*	7/8
	Wilson	2006	*	NA	*	*	**	*	*	*	8/8
Case-control studies			*Case definition*	*Representativeness of the cases*	*Selection of controls*	*definition of controls*	*Comparability of cases and controls on the basis of the design or analysis*	*Ascertainment of exposure*	*same method of ascertainment for cases and controls*	*non-response rate*	number of stars awarded/ maximum number of stars
	Khamis	2016		*	*	*	**	*	NA	NA	6/7
	Ravendi	2011		*	*	*	**	*	NA	NA	6/7
	Van den Berg	2018		*	*	*	**	*	NA	NA	6/7
Cross-sectional studies			*Representativeness of the sample*	*Sample size*	*Non-respondents*	*Ascertainment of the exposure (risk factor)*	*Comparability of subjects in different outcome groups*	*Assessment of the outcome*	*Statistical test*		number of stars awarded/ maximum number of stars
	Bilgen	2005	*		NA	**					3/9
	Che	2011	*		NA	**			*		4/9
	Chen	2011	*		NA	**	*				4/9
	Garrido-Sanchez	2009	*		NA						1/9
	Gruzdeva	2014	*		NA	**					3/9
	Maiolino†	2012	*		NA	**					3/9
	Moohebati	2014	*		NA	**					3/9
	Rossi	2003	*	*	NA	**	*	**			7/9
	Soto	2009	*		NA	**		*			4/9
	Tsimikas†	2007	*		NA	**	*		*		5/9

** Comparability can be rated with two stars*
† Article applied both cohort design and cross-sectional design
